# Supplementary figures and images for: Effects of ACTH-Induced Long-Term Hypercortisolism on the Transcriptome of Canine Visceral Adipose Tissue
Source: Vet Sci. 2022 May 25;9(6):250. doi: 10.3390/vetsci9060250 (PMC9228614; doi:10.3390/vetsci9060250)

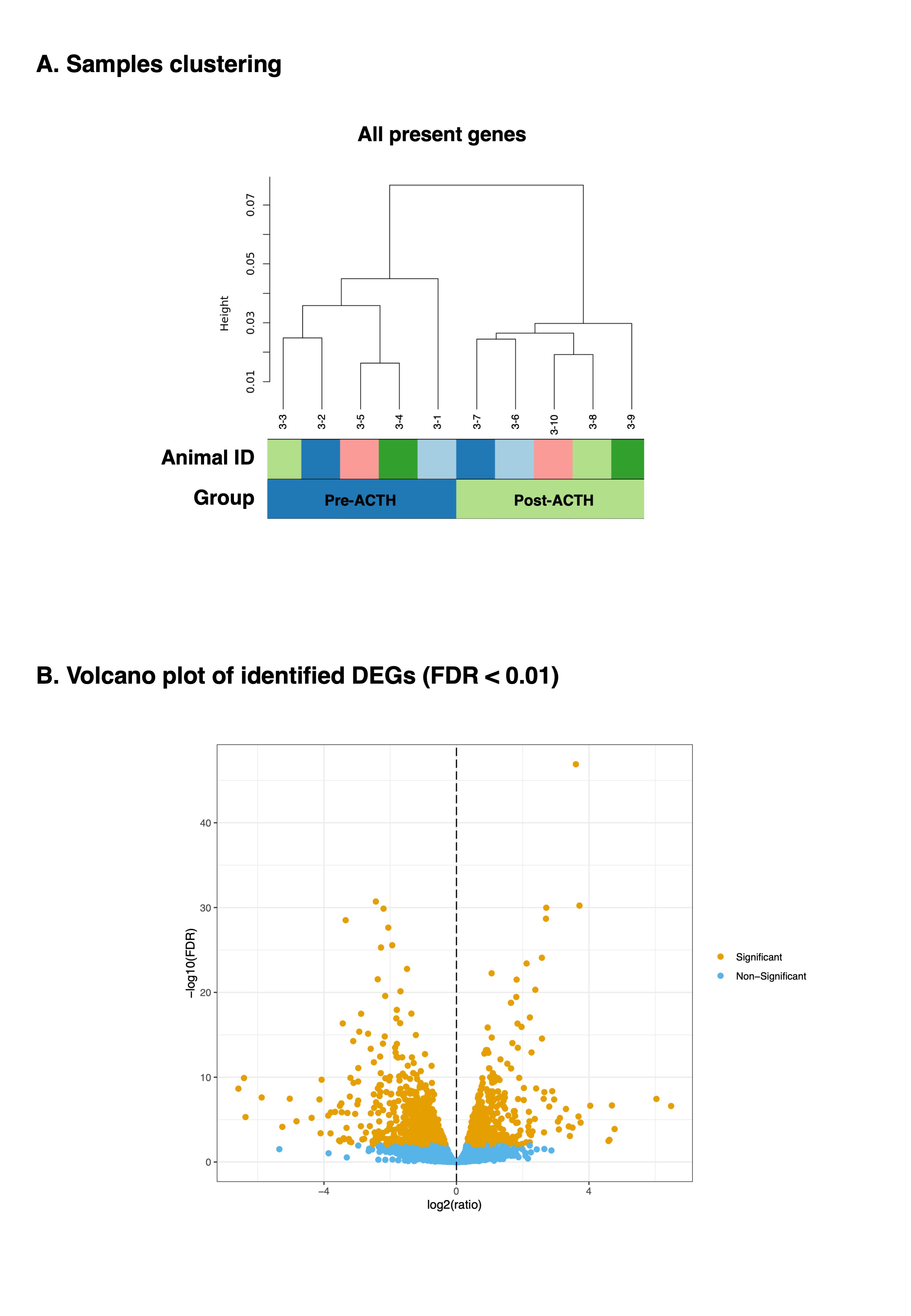

Supplement: Supplementary file 1 [file vetsci-09-00250-s001.zip › Figure S1.jpeg]
